# Changing Management of Hematological Malignancies With COVID-19: Statement and Recommendations of the Lebanese Society of Hematology and Blood Transfusion

**DOI:** 10.3389/fonc.2021.564383

**Published:** 2021-03-15

**Authors:** Ahmad Ibrahim, Peter Noun, Charbel Khalil, Ali Taher

**Affiliations:** ^1^Division of Hematology-Oncology, Department of Medicine, Lebanese and Arab Universities, Beirut, Lebanon; ^2^Cancer Center and Bone Marrow Transplantation (BMT) Program at Middle East Institute of Health, Bsalim, Lebanon; ^3^Bone Marrow Transplantation (BMT) Program at Makassed University Hospital, Beirut, Lebanon; ^4^Lebanese Society of Hematology and Blood Transfusion, Beirut, Lebanon; ^5^Division of Pediatrics, Balamand University, Beirut, Lebanon; ^6^Division of Pediatric Hematology-Oncology, Saint Georges University Medical Center, Beirut, Lebanon; ^7^Bone Marrow Transplantation (BMT) Program at Middle East Institute of Health University Hospital, Beirut, Lebanon; ^8^Faculty of Pharmacy, Saint Joseph University, Beirut, Lebanon; ^9^Division of Hematology-Oncology, Department of Medicine, American University of Beirut, Beirut, Lebanon; ^10^Cancer Center of the American University of Beirut, Beirut, Lebanon

**Keywords:** SARS-Cov-2 infection, LSHBT, hematological malignancies, myeloid, lymphoid

## Abstract

COVID-19 caused by SARS-Cov-2 is a devastating infection in patients with hematological malignancies. In 2018, the Lebanese Society of Hematology and Blood Transfusion (LSHBT) updated the guidelines for the management of hematological malignancies in Lebanon. In 2019, it was followed by a second update. Given the rapidly changing evidence and general situation for COVID-19, the LSHBT established some recommendations and suggestions for the management of the patients with hematological malignancies taking into account the Lebanese condition, economic situation, and the facts that SARS-Cov-2 infection has apparently been devastating. In this article we present recommendations and proposals to reduce or to manage SARS-Cov-2 infection in the patients with myeloid and lymphoid hematological malignancies.

## Introduction

With almost 101.5 million of declared infected persons including at least 2.18 million deaths, COVID-19 caused by SARS-Cov-2 is a devastating infection particularly in patients with hematological malignancies (1).

In Lebanon with almost 290K persons were detected positive and 2,553 died, the novel corona virus pandemic which seem to be less devastating is spreading (2).

Patients with hematological malignancies are treated in Lebanon in different types of structures having a heterogeneous expertise, including public and private academic and non-academic hospitals, military hospitals and clinics. On the other hand, coverage of the management is heterogeneously assured by the Lebanese ministry of public health, the national social security system, the army, the national cooperative system and the private insurances. Do note that almost 10–15% of the patients have to pay to be treated. Currently, there are four bone marrow transplantation units including three which perform allogeneic and autologous transplants and are European Society for Blood and Marrow Transplantation (EBMT) members and one which only performs autologous transplantation. CART-cell therapy is still not available in the country.

The Lebanese Society for Hematology and Blood Transfusion (LSHBT) presented in 2018 to the Lebanese ministry of public health which updated in 2019, with the help of United Nations Development program/TOKTEN (UNDP). An update of national guidelines for hematological diseases including hematological malignancies in order to harmonize patients management (3). In view of the rapidly changing evidence and general situation for COVID-19, the LSHBT proposed some recommendations and suggestions for the management of the patients with hematological malignancies in the era of SARS-Cov-2 infection. These recommendations collected from different international societies guidelines (4–8) were adapted to the Lebanese condition.

The aim of this approach is to assist Lebanese hematologists and oncologists in their clinical decision making and management of the patients; but, it remains the responsibility of each physician to decide the appropriate medical management and advice for the patients.

In this article we present recommendations and proposals to reduce or to manage SARS-Cov-2 infection in the patients with myeloid and lymphoid hematological malignancies.

## General Recommendations

A strict triage upon access to care must be done. COVID-19 nasopharyngeal swab of the patients must be performed and results obtained before admission to the hematology unit. It is recommended to avoid sending a possible COVID-19 patient, particularly immunocompromised, to emergency room. In case of urgent admission, care should be taken similarly to patient infected in a specific COVID-19 unit until obtaining the result of PCR. If PCR is negative, the patient can be transferred to the hematology unit. Symptomatic and/or PCR positive patients must be admitted to a specific COVID-19 unit in close collaboration with ICU. So if possible, it is necessary to create a specific hematology unit for COVID-19 positive patients with dedicated personnel.

Patients should avoid getting infected by strict adherence to hand hygiene, mask use (surgical, FFP2/3), physical distancing and home isolation. It is preferable to educate carefully and provide written interactive measures.

It is recommended to avoid crowded clinics by deferring regular routine follow-up and particularly over-the phone consultations, telemedicine or telehealth if available.

It is also necessary to consider limitation of hospital admissions or visits by preferring oral or S/C medications or delaying treatment if possible.

Visits of the patients in the hospital must be reduced at maximum or avoided if possible.

Because important effort should be done in order to prevent COVID-19 in healthcare workers, appropriate distancing and use of mask are necessary, so it is recommended to educate the staff on the use of PPE. Swab for SARS-Cov-2 must be done for any symptomatic healthcare worker or exposed to SARS-Cov-2 positive person. If any symptom appears in any healthcare worker, he or she should stay home isolated.

Inclusion in clinical trials should be postponed if possible and follow-up of patients included should be modified in order to reduce hospital visits and tests.

In Lebanon, classical cytogenetic and basic molecular testing is available and affordable for the majority of the patients with hematological malignancies. Flow cytometry for diagnosis and residual disease analysis is also available and done for quasi all patients. Next generation sequencing can be performed in the country but still not routinely performed in the clinical practice. It is necessary to develop these genomic analyses which are helpful for diagnosis, prognosis and management of the patients with hematological malignancies in order to facilitate the decision of the appropriate strategy for induction and consolidation therapy particularly in the SARS-Cov-2 pandemic (9, 10).

In Lebanon, drug are provided to the patients by the hospitals, but also by other structures like ministry of health, the army and related, the cooperative system etc… practically on monthly basis. So, it is necessary to create a homogenous system in order to deliver drugs for 2 or 3 months to limit patient's visits.

## Recommendations to Reduce SARS-Cov-2 Infection in Myeloid Hematological Malignancies

### Acute Myeloblastic Leukemia

Induction and consolidation therapy in Acute Myeloblastic Leukemia (AML) patients could induce infectious complications because of severe and prolonged neutropenia. Infections are in most cases due to bacterial and/or fungal microorganisms; however, viral infections could occurred, in particular due to respiratory virus, with well-documented cases of severe and fatal respiratory infection in SARS-Cov-2 hematologic patients. So there is a need for changing therapeutic approach in some patients.

Table 1 summarized the recommendations for AML.

**Table 1:** Acute myeloblastic leukemia.

Testing	• For newly diagnosed patients • If fever and/or other symptoms related to SARS-Cov-2 infection • Donor and recipient before allogeneic transplant
Induction (AML non-APL)	• Consider starting HU in proliferative AML which helps to delay induction if necessary • **Eligible patients:** 3 + 7 regimen • **Non-eligible patients:** hypomethylating agents (±Venetoclax) • Targeted therapy for FLT3 or IDH 1/2 mutations should be given • Mylotarg (Anti CD33) for CBF AML patients • G-CSF to shorten neutropenia • Lowering transfusion episodes, by lowering thresholds for Hb (<7 g/dl) and platelets (<10 × 10^9^/L)
Consolidation (AML non-APL)	• **Eligible patients in CR** after induction: reduction of the doses and cycles of cytosine-arabinoside • **Non-eligible patients in CR** after induction: hypomethylating agents (±Venetoclax) • G-CSF and transfusion as for induction
Relapsed/refractory (AML non-APL)	• **Eligible patients:** salvage intensive re-induction therapy • **Non-eligible patients:** and if non-proliferative AML, consider hypomethylating agents (±Venetoclax) • Consider starting HU in proliferative AML • Targeted therapy (anti FLT3 or IDH1/2) should be given • G-CSF and transfusion as for induction
APL	• **Lower-risk:** ATRA and ATO as per standard treatment • **High-risk:** ATRA and ATO added to cytoreductive chemotherapy as per standard treatment • If ATO not available, ATRA and anthracyclines
Allogeneic transplant	• No delay

#### Testing for SARS-Cov-2

Testing should be performed for all newly diagnosed AML patients, and at the start of the next treatment cycle. Patients with fever and/or other symptoms related to SARS-Cov-2 infection should have an additional test at any time. Donor and recipient for allogeneic transplant should also be tested before the procedure.

#### Induction Therapy for AML (Non-APL)

Intensive induction chemotherapy should still be offered for eligible patients with 3 + 7 (anthracyclines for 3 days and cytosine- arabinoside for 7 days) or similar regimens (11). Fludarabine combination should be avoided because of the deep immunosuppression effect of this drug. For the other patients, non-chemotherapeutic induction could be hypomethylating agents with or without low doses of cytosine-arabinoside (12). Adding of venetoclax to hypomethylating agents can be considered but at lower doses of venetoclax because the combination could induce long phases of aplasia (12–14). This combination can also be administered as alternative to chemotherapy in patients with positive NPM1 mutation because of high rate of durable complete remission (CR) obtained (15). Furthermore, venetoclax based regimens can be given for any non-CBF elderly patient (>60 year-old), or patient over 50 year-old with MPN1 or IDH1/2 mutations (14).

Mylotarg (antiCD33) can be administered to CBF AML patients in combination with 3 + 7 regimen due to the significant improvement of survival in these patients with this combination. Myelotarg is omitted from chemotherapy combination for intermediate- and high-risk patients (16, 17).

Other targeted therapies (e.g., Anti FLT3 or anti IDH1/2) when indicated should be administered.

#### Consolidation Therapy for AML (Non-APL)

For eligible patients with CR after induction chemotherapy, consolidation with high-dose cytosine-arabinoside based regimens should be offered, but reducing the dose of cytosine-arabinoside (e.g., 1.5/m^2^ instead of 3 g/m^2^ per dose) and decreasing the number of cycles to 2–3 instead of four are considered, since randomized studies showed disease-free-survival (DFS) advantage only in favorable cytogenetic risk patients at higher doses (3 g/m^2^) of cytosine-arabinoside (18). For the other patients, hypomethylating agents could be given.

#### Relapse/Refractory AML (Non-APL)

Standard intensive regimens are used for eligible patients. For the other patients, and if the disease is not proliferative re-induction therapy could be temporarily postponed. Hydroxyurea (HU) could be given if the disease in proliferation awaiting re-induction therapy.

Targeted therapy (hypomethylating agents, venetoclax, anti FLT3 or anti IDH1/2, etc…) can be administered.

#### Supportive Care

Because of difficulties and possible shortages in blood bank supplies, lowering transfusion episodes, by lowering transfusion thresholds (e.g., 7 g/dL for Hb, 10 × 10^9^/L for platelets) should be considered for patients without symptoms due to anemia or thrombocytopenia. G-CSF can be administered to shorten neutropenia.

#### Allogeneic Hematopoietic Stem Cell Transplantation

Allogeneic transplant should not be delayed. Cryopreservation of donor cells prior to starting the procedure should be considered.

#### Acute Promyelocytic Leukemia

All patients with low-risk APL should be treated when available with all transretinoic acid (ATRA) and arsenic trioxide (ATO) as per standard treatment. For high-risk patients, ATRA and ATO added to cytoreduction therapy should be given as per standard regimens. If ATO is not available, ATRA could be added to chemotherapy (e.g., anthracycline) (19).

### Myelodysplastic Syndromes

Patients with Myelodysplastic Syndromes (MDS) who are lymphopenic or neutropenic or have undergone allogeneic transplant are at high-risk after contracting SARS-Cov-2 to get serious complications.

Table 2 summarized the recommendations for MDS.

**Table 2:** Myelodysplastic syndromes.

Testing	• For newly diagnosed patients • If fever and/or other symptoms related to SARS-Cov2 infection • Donor and recipient before allogeneic transplant
Initial therapy	• **Higher-risk:** hypomethylating agents (±venetoclax) • **Lower-risk:** agent to reduce transfusion needs (ESA, lenalidomide) • **Del (5q) syndrome:** lenalidomide • Lowering transfusion episodes if possible, by lowering the threshold for Hb (<7 g/dl) and platelets (<10 × 10^9^/L) • G-CSF to shorter neutropenia • Transfusion as for AML
Relapsed/refractory	• Management on a case-by-case basis
Allogeneic transplant	• No delay

#### Testing for SARS-Cov-2

Testing should be performed for all newly diagnosed patients, then at the start of the next treatment cycle. Patients with fever and/or other symptoms related to SARS-Cov-2 infection should have an additional test at any time. Donor and recipient for allogeneic transplant should also be tested before starting the procedure.

#### Initial Therapy

Patients with high-risk MDS can receive hypomethylating agents with or without venetoclax, without dose adjustment (20).

Patients with lower-risk MDS, therapy that reduces transfusion needs (erythropoiesis stimulating agents, lenalidomide…) should be used (20). Luspatercep, an erythroid maturation agent, not available currently in Lebanon, but approved by the FDA for the treatment of anemia failing an erythropoiesis stimulating agent and requiring two or more red blood cell (RBC) units over 8 weeks in adult patients with very low- to intermediate-risk MDS with ring sideroblasts (MDS-RS) or with MDS/MPNs with ring sideroblasts and thrombocytosis (MDS/MPN-RS-T), can be used if the patient has the drug on his or her own (21).

For patients with del (5q) syndrome, lenalidomide can be given (20).

Because of difficulties and possible shortages in blood bank supplies, lowering transfusion episodes by lowering transfusion thresholds (e.g., 7 g/dl for Hb, 10 × 10^9^/L for platelets) should be done for patients without symptoms due to anemia or thrombocytopenia. G-CSF can be given to shorten neutropenia.

#### Relapsed/Refractory Patients

Therapy should be decided on a case-by-case basis.

#### Allogeneic Hematopoietic Stem Cell Transplantation

Efforts should be made not to delay admission for allogeneic transplant. Cryopreservation of donor cells prior to the procedure is preferable.

### Chronic Myelogenous Leukemia

Table 3 summarized the recommendations for Chronic Myelogenous Leukemia (CML).

**Table 3:** Chronic myelogenous leukemia.

Testing	• For newly diagnosed patients • If fever and/or other symptoms related to SARS-Cov2 infection • Donor and recipient before allogeneic transplant
Initial therapy	• No delay • No change in TKIs monitoring
Resistance/Intolerance to TKI	• No delay in changing TKIs • No change in monitoring
Treatment-free-remission	• If started, possible reduction in monitoring schedules (every 1.5 months the first 6 months, then every 2–3 months. • If no possibility to perform PCR adequately, postponed TFR and restart TKI
Accelerated phase	• No change if response to TKIs
Blast crisis	• Intensive TKI-based therapy
Allogeneic transplant	• No delay

#### Testing for SARS-Cov-2

Testing should be performed for all newly diagnosed CML patients. Patients with fever and/or other symptoms related to SARS-Cov-2 infections should have an additional test at any time. Donor and recipient for allogeneic transplant should also be tested before starting the procedure.

#### Chronic Phase

Neither chronic phase CML nor BCR-ABL tyrosine kinase inhibitors (TKIs) induce significant immune suppression and there has been no data suggesting that CML patients in first chronic phase may be at higher risk of infection by the novel coronavirus than the general population. Based on that, newly diagnosed CML treatment choice does not need to change with no delay of introduction of TKIs; however, during the first 3 months of TKI therapy, extreme caution is advised because myelosuppression may occur in some patients and regular control of blood count (every 1–2 weeks) is recommended. In order to avoid severe pancytopenia, in particular neutropenia, dose interruptions or reductions should be done on a case- to case basis (22).

For CML patients already on therapy with TKIs, treatment should not be changed because interruption of TKIs may lead to loss of response and progression or relapse (22).

Patients in remission on regular PCR monitoring should continue this schedule. To minimize patient travel and exposure, remote testing (test done at home) is to be considered.

Careful attention should be made to metabolic, cardiovascular or pulmonary side effects induced by some TKIs which might complicate SARS-Cov-2 infection (19).

In patients facing resistance or intolerance to current TKI, it is not recommended to delay a change in therapy as outcome may be worsened (22).

Patients who have entered treatment-free-remission (TFR) for <6–12 months and requiring more frequent monitoring, as often as monthly, have to continue if it is possible; however, a decrease in frequency can be considered every 1.5–2 months in the first 6 months then every 2–3 months in case of difficulties to perform the monthly monitoring required. The patients who do not have access to regular monitoring of blood count and BCR-ABL transcripts are advised to postpone TFR and restart TKI treatment (22).

#### Accelerated Phase and Blast Crisis

Patients with accelerated phase CML responding well to TKI therapy should continue with proper monitoring.

Younger patients with blast crisis may be considered for intensive TKI based therapy in centers allowing for proper monitoring and support.

#### Allogeneic Hematopoietic Stem Cell Transplantation

Efforts should be made not to delay admission for allogeneic transplant if indicated. Cryopreservation of donor cells prior to the procedure is preferable.

### Myeloproliferative Neoplasms

Elderly patients with a Myeloproliferative Neoplasm (MPN), particularly with additional illness such as cardiovascular or thrombotic disorders and diabetes are considered more vulnerable and less able to recover from SARS-Cov-2 infection.

Table 4 summarized the recommendations for MPNs.

**Table 4:** Myeloproliferative neoplasms.

Testing	• For newly diagnosed patients • If fever and/or other symptoms related to SARS-Cov2 infection • Donor and recipient before allogeneic transplant
Initial therapy	• **PV:** Phlebotomy and Aspirin No adjustment of cytoreductive therapy • **ET:** No adjustment of cytoreductive therapy • **PMF:** ruxolitinib for intermediate or higher risk patients. Monitoring of blood count and coagulation parameters (risk of bleeding or thrombosis)
Allogeneic transplant for PMF	• No delay

#### Testing for SARS-Cov-2

Testing should be performed for all newly diagnosed MPNs patients, and if fever and/or other symptoms related to SARS-Cov-2. Donor and recipient for allogeneic transplant should also be tested before starting the procedure.

#### Treatments

There is currently no evidence that elderly patients, with essential thrombocythemia (ET) or polycythemia vera (PV) who are on anticoagulants are at increased risk of SARS-Cov-2 infection compared with the general population. Some treatments for MPNs or co-morbid cardio vascular thrombotic or cardiovascular disorders may decrease the ability to recover from SARS-Cov-2 infection. Patients with intermediate-2 or high-risk primary myelofibrosis (PMF) on JAK2 inhibitors could have a worse outcome if they become infected with the virus; however, the effect of JAK inhibitors increasing of SARS-Cov-2 infection is unknown (23). On the other hand, the abrupt cessation of JAK2 inhibitor (ruxolitinib) may result in progressive disease or rarely cytokine storm which worsens the clinical course of SARS-Cov-2 infection, and it has been suggested that ruxolitinib it can be a possible therapy for cytokines storm in critically ill COVID-19 patients (24). Given the base line increased risk and frequenting of thromboembolic complications in MPNs and advanced stage COVID-19 patients, it is necessary to reduce hematocrit <45%. And to maintain a WBC <10 × 10^9^/L and platelet <400 × 10^9^/L. Cytoreductive therapy should be maintain if there is a history or an active thromboembolic or hemorrhagic event (25).

Patients on phlebotomy for PV can maintain it but decreasing the frequency is considered. Because of unclear situation with MPNs, cytoreduction (hydroxyurea, IFN, anagrelide….) should not be adjusted. For PMF patients the management must be established according to know prognostic factors which can be evaluated by MIPSS or DIPSS plus or equivalent (26). JAK2 inhibitors therapy such as ruxolitinib could be given if clinical situation allows. If ruxolitinib needs to be stopped, careful tapering must be done (25).

#### Allogeneic Hematopoietic Stem Cell Transplantation for PMF

Efforts should be made not to delay admission for allogeneic transplant. Cryopreservation of donor cells prior to the procedure is preferable.

## Myeloid Hematological Malignancies With SARS-Cov-2 Infection (PCR Positive and/or Symptoms)

Following the recent update in the management of SARS-Cov-2 infection, several consideration were taken into account (4–8, 27).

### Acute Myeloblastic Leukemia

In young adults positive for SARS-Cov-2 but asymptomatic, standard induction chemotherapy with 3 + 7 or similar regimen can be given. For patients positive and symptomatic, induction therapy is postponed if the disease is low proliferative until negativation of PCR and symptoms. Awaiting, HU and hypomethylating agents with or without venetoclax can be given.

For patient in complete remission and positive for SARS-Cov-2, consolidation chemotherapy should be postponed until PCR negativity and/or resolution of symptoms. Awaiting, hypomethylating agents can be given.

Other targeted therapy (Anti-FLT3 and IDH1/2), if indicated, could be administered.

Drug interaction due to CYP3 inhibitors or potential QT prolongation between anti FLT-3, venetoclax and current therapeutic options of SARS-Cov-2 infection must be carefully taken into consideration.

Allogeneic transplant must be deferred until no symptoms and PCR negativity for at least 3–4 weeks.

### Myelodysplastic Syndromes

There is no change in specific therapy for infected patients.

Drug interaction due to CYP3 inhibitors or potential QT prolongation between specific treatment and current therapeutic options of SARS-Cov-2 infection must be carefully taken into consideration.

Allogeneic transplant must be deferred until no symptoms and PCR negativity for at least 3–4 weeks.

### Chronic Myelogenous Leukemia

If case of non-severe infection, interruption of TKIs therapy is not recommended except if the patient has cardio pulmonary toxicity due to TKIs.

TKIs are interrupted in case of severe infection.

Patient in TFR should be managed the same way as the general population.

Allogeneic transplant should be deferred until no symptoms and PCR negativity for at least 3–4 weeks.

### Myeloproliferative Neoplasms

The patients should be started or switched to heparin prophylactically. In case of massive or sub-massive pulmonary embolism which should be suspected in case of respiratory deterioration and a sudden increase of D-Dimer, thrombolysis should be considered.

Cytoreductive therapy (HU, IFN, anagrelide) should not be stopped or empirically adjusted.

Dose of ruxolitinib should be modified if an antiviral therapy (lopinavir, ritonavir) is used for SARS-Cov-2 infection.

Careful monitoring of blood counts and coagulation parameters, in particular with anticoagulation to intercept thrombocytopenia or coagulation abnormalities, must be done because of high risk of thrombosis and bleeding.

Allogeneic transplant should be deferred until resolution of symptoms and PCR negativity for at least 3–4 weeks.

## Recommendations to Reduce SARS-Cov-2 Infection in Lymphoid Hematological Malignancies

### Acute Lymphoblastic Leukemia

Table 5 summarizes the recommendations for Acute Lymphoblastic Leukemia (ALL).

**Table 5:** Acute lymphoblastic leukemia.

Testing	• At diagnosis • Before every cycle • If fever and/or other symptoms related to SARS-Cov-2 infection • Donor and recipient before allogeneic transplantation
Induction	• **Ph negative** ° Can be done according to standard regimens but lower doses of steroids, lower doses of anthracyclines if and lower doses of asparaginase ° No anti CD20 if IgG is low ° Light chemotherapy and antibodies (inotuzumab, blinatumomab) • **Ph positive** ° Consider Tyrosine Kinase Inhibitors (TKis) and steroids with light chemotherapy (vincristine) without multiagent chemotherapy • G-CSF to shorten neutropenia
Intensification post-remission	• Modifications to reduce admissions and prolonged neutropenia
Maintenance therapy	• On schedule, but lower doses of steroids (maintain ANC ≥1 × 10^9^/L)
Relapsed/refractory	• B-ALL: antibodies (inotuzumab, blinatunomab) preferred to multiagent chemotherapy • T-ALL: according to standard regimens but dose reduction
Allogeneic transplantation	• No delay

#### Testing for SARS-Cov-2

Testing is recommended at diagnosis and at initiation of every cycle of therapy. It should be done if fever and/or other symptoms related to SARS-Cov-2 infection at any time. Donor and recipient are tested before allogeneic transplantation.

#### Induction Therapy

For the patients with ALL Ph-negative, standard induction therapy can be done because delay is associated with poor outcome; however, lower doses of steroids should be given. Reduction of anthracyclines doses is preferred in the patients at high-risk complications if SARS-CoV-2 is contracted. Because of the changes in coagulation parameters and thromboembolic complications induced by the virus, reduction of the dose of asparaginase is recommended. Anti CD20, if indicated, could be skipped; however, if IgG level is adequate and no infection, it administration is considered.

Because of the good results of anti CD22 (inotuzumab ozogamycin), in combination with low-intensity chemotherapy [e.g., minihyperCVD (cyclophosphamide, vincristine, dexamethasone)] for older patients with ALL Ph-negative in first line therapy, this approach could be considered as an alternative, but it depends on the availability and the cost of anti CD22. Blinatumomab, a specific antibody anti-CD3/CD19, based combination could be also considered (28, 29).

For the patients with ALL Ph positive, using tyrosine kinase inhibitors (TKIs) with steroids and vincristine instead of multiagent chemotherapy should be considered (30).

G-CSF should be given to shorten neutropenia.

#### Post-remission Therapy

Delay of intensification course is preferable, however, if started, some modifications of intensive post-remission therapy should be done to reduce hospital admissions and/or prolonged immunosuppression and neutropenia. For example, cytarabine could be given s/c at home, and anti CD20 given only if IgG level is normal.

Maintenance therapy can be given on schedule but with a reduction in steroid doses, and absolute neutrophil count (ANC) should be maintained at ≥1 × 10^9^L.

G-CSF should be given to shorten neutropenia.

#### Relapse/Refractory ALL

For patients with B-ALL, specific antibodies (inotuzumab or blinatumomab) are preferable to other regimens (28).

For patients with T-ALL, salvage regimens according to standard regimens but with possible doses reduction can be given (28).

#### Allogeneic Hematopoietic Stem Cell Transplantation

For patients with high-risk ALL, allogeneic transplant can be performed in first remission, but taking into account the risks/benefits. Patients who achieve a second complete remission should undergo allogeneic transplant despite pandemic. Cryopreservation of donor cells before starting the procedure should be considered.

### Chronic Lymphocytic Leukemia

Table 6 summarizes the recommendations for Chronic Lymphocytic Leukemia (CLL).

**Table 6:** Chronic lymphocytic leukemia.

Testing	• At diagnosis • If fever and/or other symptoms related to SARS-CoV-2 • Donor and recipient before allogeneic transplantation
Newly diagnosis	• If immediate therapy is required, avoid monoclonal antibodies (anti CD20) and anti Bcl 2 (Venetoclax) • IVIG should be given for patients with hypogammaglobulinemia and recurrent infections, but less frequently (every 6–8 weeks)
On therapy	• Skip or delay monoclonal antibodies • IVIG should be given for patients with hypogammaglobulinemia and recurrent infection, but less frequently (6–8 weeks)
Allogeneic transplantation	• Delay if stable disease

#### Introduction

Patients with CLL are at higher risk of contraction of severe SARS-Cov-2 infection than general population because of underlying immunodeficiency and inadequate immune response to infection.

#### Testing for SARS-Cov-2

Testing should be performed for all newly diagnosed patients and if fever and/or other symptoms related to SARS-Cov-2 infection at any time. Donor and recipient should be tested before starting allogeneic transplantation.

#### Treatment

For newly diagnosed patients, if immediate therapy is required, treatment with monoclonal antibodies (anti CD20) should be avoided or skipped. BCR signaling inhibitors can be given, but treatment with anti Bcl-2 (venetoclax), which requires at its initiation multiple visits and lab tests, should be delayed or avoided.

Immunoglobulins (IVIG) should be given for patients with hypogammaglobulinemia and severe or recurrent infections, but infusion of IVIG could be done less frequently (every 6–8 weeks).

For the patients already on therapy, no significant changes are required except for monoclonal antibodies (anti CD20) and for IVIG, which must be similar to newly diagnosed patients (31).

#### Hematopoietic Stem Cell Transplantation

Delaying allogeneic transplantation should be considered if the disease is stable. Cryopreservation of donor cells should be considered before starting the procedure.

### Aggressive Lymphomas

#### Diffuse Large B-Cell Lymphoma

Table 7 summarizes the recommendations for Diffuse Large B-Cell Lymphoma (DLBCL).

**Table 7:** Diffuse Large B-cell NHL.

Testing	• At diagnosis • Before every cycle • If fever and/or other symptoms related to SARS-Cov-2 infection • Before mobilization and before autologous transplantation • Donor and recipient before allogeneic transplantation
Initial therapy	• **Younger patients:** ° R-CHOP ° CNS prophylaxis to high-risk groups (testicular, renal, adrenal) ° CNS involvement: intrathecal chemotherapy/hig-dose methotrexate ° More intensive {[DA-EPOCH-R (rituximab, etoposide phosphate, prednisone, vincristine sulfate (Oncovin), cyclophosphamide, and doxorubicin hydrochloride) or R-ACVBP (adriamycin, cyclophosphamide, vindesine, bleomycin, prednisone, rituximab)]} for high-risk or double hit ° Delay radiotherapy • **Elderly patients:** ° R-mini CHOP • G-CSF to minimize neutropenia
Relapse/refractory	• Salvage therapy according to standard protocols • Avoid T-cell suppressive agents (bendamustine) • Consider outpatient salvage regimens (rituximab, revlimid ± anti CD-20) • Anti CD796 based therapy • G-CSF to minimize neutropenia
Hematopoietic stem cells transplant	• No delay for autologous transplantation in sensitive relapse • Allogeneic transplantation delayed if stable disease

##### Testing for SARS-Cov-2

Testing should be performed for all newly diagnosed patients, and at the start of treatment cycle. Patients with fever and/or other symptoms related to SARS-Cov-2 infection should have an additional test at any time. All patients should be tested before mobilization of peripheral blood stem cells and before autologous transplantation. Donor and recipient for allogeneic transplant should also be tested before the procedure.

##### Initial Therapy

For younger patients R-CHOP (rituximab, cyclophosphamide, doxorubicin hydrochloride, vincristine, and prednisone) every 3 weeks is the preferable regimen; however, intensive but more toxic therapy such as DA-EPOCH-R (etoposide, prednisone, vincristine, cyclophosphamide, doxorubicin, rituximab) or R-ACVBP (doxorubicin, cyclophosphamide, vindesine, bleomycin, prednisone, and rituximab) for high-risk or double hit could be administered if the center has the capacity to deliver it. In this case, patients should receive G-CSF to minimize neutropenia (32).

If there is a CNS involvement, high-dose methotrexate (for example 2 cycles) and intrathecal chemotherapy can be given.

For older patients, R-mini CHOP with G-CSF is recommended (32).

##### Relapse/Refractory DLBCL

Salvage therapy according to standard regimens could be given with close follow-up and administration of G-CSF to minimize neutropenia. Anti CD79 antibody (polatuzumab) in combination with anti CD20 and chemotherapy can also be administered (33). T-cell suppressive agents (example bendamustine) should be avoided. If the center cannot handle these salvage regimens, outpatient therapy including oral targeted therapy (e.g., lenalidomide) with anti CD20 is an alternative (34).

##### Hematopoietic Stem Cell Transplantation

Autologous peripheral blood stem cells transplantation, mainly indicated in sensitive relapse, can be performed.

Allogeneic hematopoietic stem cells transplantation can be delayed if stable disease. Cryopreservation of donor cells should be considered before starting the procedure.

#### Mantle Cell Lymphoma

For fit patients, first line therapy according to standard protocols including anti-CD20 may be given with dose reduction.

For elderly patients, R-CHOP, or R-mini CHOP should be used.

Local radiotherapy and steroids for symptomatic localized disease should be considered.

Systemic therapy for relapsed/refractory disease may be reduced in intensity or replaced by oral targeted based regimens (lenalidomide, BCR inhibitors like ibrutinib) (35).

T-cell suppressive agents (e.g., bendamustine) should be avoided.

Anti CD20 (rituximab) can be administered in first and subsequent lines; however, maintenance therapy with rituximab may be delayed.

For transplantation, strategy is similar to DLBCL.

G-CSF support to minimize neutropenia should be used.

##### Primary Mediastinal B-Cell Lymphoma

R-CHOP-14 and omitting radiotherapy if PET negative should be considered.

More aggressive chemotherapy (e.g., DA-EPOCH-R) with omission of radiotherapy if PET negative should be considered for selected patients (36).

For relapse/refractory and transplant, the strategy is similar to DLBCL.

##### Burkitt's Lymphoma

First line therapy according to standard protocols can be given with a consideration to reduce methotrexate and cytarabine doses (37).

##### Primary CNS Lymphoma

For first line therapy, reduction of the dose of cytarabine per cycle should be considered. Thiotepa should be omitted for elderly patients (38).

For autologous transplant as consolidation therapy, reduction of the doses of thiotepa should be done.

##### Peripheral T-Cell Lymphoma

For first line therapy, CHOP with G-CSG support can be given; however, standard protocols can also be given if the center can handle them.

Autologous transplant as consolidation can be delayed if stable disease, otherwise for autologous and allogeneic transplant, the strategy is similar to DLBCL.

For relapse/refractory patients, management can be decided on case-by-case. If indicated, anti CD30 (brentuximab vedotin) combination can be given (39).

##### Cutaneous T-Cell Lymphoma

Patients with primary cutaneous lymphoma (PCL) tend to be older and remain on long-term immunosuppressive therapy for disease control which can contribute to the development of more severe complications for SARS-Cov-2 infection.

For low-risk PCL patients, local therapies that can be utilized at home should be continued (e.g., topical retinoids, topical steroids, home UVB heliotherapy). Electron beam radiation therapy and ultraviolet light therapy should be delayed.

For intermediate-risk patients, treatment with oral retinoids, oral methotrexate, oral steroids, interferon (IFN), vorinostat may be continued with dose adjustment which allows limiting hospital visits and lab monitoring.

For high-risk patients, infusion regimens (parotrexate, romidepsin, brentuximab vedotin, gemcitabine, etc.) may be adjusted with increase to treatment intervals, particularly for romidepsin.

Other infusion regimens (CHOP, alemtuzumab, fludarabine) which can lead to significant cytopenia, should not be administered (40).

Allogeneic hematopoietic stem cells transplantation should be delayed during the pandemic.

### Indolent Lymphoma

Table 8 summarizes the recommendations for indolent lymphoma.

**Table 8:** Indolent lymphoma.

Testing	• At diagnosis • If fever and/or other symptoms related to SARS-Cov-2 infection • Before mobilization and before autologous transplantation • Donor and recipient before allogeneic transplantation
Initial therapy	• Radiotherapy for limited disease • Anti CD20 • Anti CD20-CVP (cyclophosphamide, vincristine, prednisolone) or anti CD20-CHOP (cyclophosphamide, doxorubicin hydrochloride, vincristine, prednisone) preferred to anti CD20-bendamustine • For small lymphocytic lymphoma/marginal zone/mantle cell, give oral targeted therapy (e.g., ibrutinib ± anti CD-20) • G-CSF to shorten neutropenia • IVIG for patients with low IgG levels and recurrent infections
Maintenance therapy	• Anti CD20 should not be initiated or should be stopped in elderly or younger patients with low IgG levels
Transformation	• Treatment similar to aggressive lymphoma
Relapsed/refractory	• Oral targeted therapy (ibrutinib, lenalidomide) with anti CD20 are preferred to more intensive therapy • No bendamustine based combinations
Hematopoietic stem cells transplantation	• Autologous or Allogeneic transplantation should not be delayed for the patients with transformation, and delayed for other patients with stable disease after relapse

#### Testing for SARS-Cov-2

Testing should be performed for all newly diagnosed patient and if fever and/or other symptoms related to SARS-Cov-2 infection at any time. All patients should be tested before mobilization of peripheral blood stem cells and before autologous transplantation. Donor and recipient should be tested before starting allogeneic transplantation.

#### Newly Diagnosed Patients

If immediate treatment is required, radiotherapy could be an option for limited disease. Anti CD20 monotherapy could be administered; however if a combination of anti CD20 with chemotherapy should be given, anti CD20-CVP (cyclophosphamide, vincristine, prednisone) or anti CD20-CHOP, are preferred to anti CD20-bendamustine because of the immunosuppressive properties of bendamustine.

Maintenance therapy with anti CD20 should be stopped or not initiated until end of SARS-COV-2 pandemic in older patients and in younger patients with low immunoglobulin levels (IgG).

For small lymphocytic lymphoma (SLL), marginal zone lymphoma or mantle cell lymphoma, specific oral targeted therapy could be given (e.g., ibrutinib) replacing intravenous treatment.

For patients who have started treatment and are in response, reducing the number of cycles and replacing the remaining cycles by oral less immunosuppressive therapy should be considered. If the patient on maintenance therapy is elderly or younger with low immunoglobulin levels (IgG), maintenance therapy should be stopped.

G-CSF support is recommended for neutropenic patients, particularly in case of recurrent infections, and IVIG supplementation could be given in patients with low IgG levels (41).

#### Transformation

In case of the transformation of the indolent lymphoma, treatment will follow the algorithm of aggressive lymphoma with intensive chemo-immunotherapy. Oral agents such as ibrutinib or lenalidomide with or without anti CD20 can also be considered as an alternative if the usage of intensive therapy is not possible (41).

#### Relapsed/Refractory Patients

Patients are managed on case-by-case basis (42).

#### Hematopoietic Stem Cell Transplantation

Autologous or allogeneic hematopoietic stem cell transplantation should be performed for the patient in transformation of the indolent lymphoma, but can be delayed for the other patients with a stable disease. Cryopreservation of donor cells should be considered before starting allogeneic transplantation.

### Hodgkin's Lymphoma

Table 9 summarizes the recommendations for Hodgkin's Lymphoma (HL).

**Table 9:** Hodgkin lymphoma.

Testing	• Before every cycle • If fever and/or other symptoms related to SARS-Cov-2 infection • Before mobilization and before autologous transplantation • Donor and recipient before allogeneic transplantation
• Younger patients • Early favorable	• ABVD (adriamycin, bleomycin, vinblastine, dacarbazine) × 2 and modified radiotherapy schedule • ABVD × 4 (no bleomycin for cycles 3, 4) • G-CSF to shorten neutropenia and prophylactic antibiotics
• Younger patients • Early unfavorable	• ABVD × 4 and modified radiotherapy schedule • ABVD × 6 (no bleomycin starting cycle 3) • G-CSF to shorten neutropenia and prophylactic antibiotics
• Younger patients • Advanced	• ABVD × 6 (no bleomycin starting cycle 3) • AAVD (brentuximab vedotin, doxorubicin, vinblastine, dacarbazine) (ECHELON 1) • RATHL approach but for escalation after ABVD with lower steroid doses BEACOPP (bleomycin, etoposide, adriamycin, cyclophosphamide, vincristine, procarbazine, prednisone) • G-CSF to shorten neutropenia and prophylactic antibiotic
Relapsed/refractory	• Outpatient salvage therapy (gemcitabine based, brentuximab vedotin, Anti PD1/PDL1) preferred to multiagent chemotherapy
Nodular lymphocyte predominant Hodgkin	• Radiotherapy for symptomatic sites • Single agent anti CD20 • If systemic therapy: R-CVP (cyclophosphamide, vincristine, prednisone)
Hematopoietic stem cells transplantation	• No delay for Autologous transplantation in sensitive relapse • Allogeneic transplantation delayed if stable disease

The objective for HL is to reduce patients' exposure to the hospital setting due to radiotherapy, to reduce or avoid usage of bleomycin which induces lung toxicity which is becoming problematic during this pandemic, and to delay if possible intensive chemotherapy which needs recurrent hospital admissions and induces hematological toxicities.

#### Testing for SARS-Cov-2

Testing should be performed for all newly diagnosed patients, then at the start of the next treatment cycle. Patients with fever and/or other symptoms related to SARS-Cov-2 infection should have an additional test at any time. All patients should be tested before mobilization of peripheral blood stem cells and before autologous transplantation. Donor and recipient for allogeneic transplant should also be tested before starting the procedure.

#### First Line Therapy

For patients with favorable early stage disease, two cycles of ABVD and limited radiotherapy remains an option, but a modified approach of radiotherapy reducing hospital attendances can be considered. However, an alternative to removal of radiotherapy could be four cycles of ABVD. Interim PET 2 can be maintained. If the PET 2 is negative and ABVD continued, consider omitting bleomycin for the next two cycles should be done (43, 44).

For patients with unfavorable early stage disease, four cycles of ABVD followed by radiotherapy remain an option with modifying the schedule of radiotherapy to limit patients exposure to the hospital. However, an alternative to removal of radiotherapy is to give ABVD until six cycles if interim PET 2 scan shows optional response. Omission of bleomycin after a negative PET 2 is recommended.

For patients with advanced stage disease, six cycles of ABVD with an interim PET 2 evaluation remains an option (44) and bleomycin is to be omitted in the following cycles after a negative PET 2, based on the results of RATHL study (risk-adapted therapy for HL) which showed no difference in 3 years progression-free survival (PFS) between 2 cycles of ABVD (45), followed by 4 cycles of AVD and 6 cycles of ABVD. It is also possible to consider A+AVD (brentuximab bedotin, doxorubicin, vinblastine, dacarbazine) regimen omitting bleomycin and introducing adcetris (anti CD30—brentuximab vedotin) based on the results of ECHELON-1 study which demonstrated that brentuximab vedotin (A) with AVD exhibited superior modified PFS vs. ABVD for frontline treatment for patients with stage III/IV classical HL (46), and because of the approval in Lebanon of A + AVD for first line therapy for stage IV HL. Do note that this approach is PET 2 independent; however, more expensive and needs G-CSF prophylactically to prevent at maximum febrile neutropenia without forgetting peripheral neuropathy induced by A + AVD.

In case of positive interim PET 2 scan, treatment intensification can be done as offered in RATHL, GITIL/FIL HD 0607 (47) and SWOG s0816 studies (48); however, four cycles of BEACOPP (bleomycin, etoposide, doxorubicin, cyclophosphamide, vincristine, procarbazine and prednisolone) should be considered instead of BEACOPP escalated.

For patients with nodular lymphocyte predominant HL, radiotherapy on symptomatic sites can be done. Single agent anti CD-20 can also be given. If systematic therapy is to be given, R-CVP should be used (43).

#### Relapse/Refractory HL

Outpatient salvage regimens are preferred to regimens that require recurrent and longer hospitalization, for example gemcitabine-based or brentuximab vedotin or anti PD1/PDL1 (nivolumab, pembrolizumab) (49).

#### Elderly Patients

All elderly patients (>60 yo) can receive treatment according to standard regimens but at reduced doses. Bleomycin should be omitted from therapy.

#### Supportive Care

G-CSF can be given 3–5 days at mid cycle of chemotherapy to reduce severe neutropenia. Prophylactic antibiotherapy for expected neutropenic patients could be given.

#### Hematopoietic Stem Cell Transplantation

Autologous peripheral blood stem cell transplantation usually used as consolidation after salvage therapy can be performed.

Allogeneic stem cell transplantation which is not commonly used in HL and usually indicated for relapse after autologous transplant, can be delayed if stable disease. Cryopreservation of donor cells should be considered before starting the procedure.

### Multiple Myeloma

Table 10 summarizes the recommendations for Multiple Myeloma (MM).

**Table 10:** Multiple myeloma.

Testing	• At diagnosis • If fever and/or other symptoms related to SARS-CoV-2 • Before mobilization and before autologous transplantation
Initial therapy	• No for patients without CRAB or smoldering MM or MGUS • **If immediate therapy indicated for transplant eligible:** ° Triplet therapy [VRd (revlimid, velcade, dexamethasone) preferred to VTD (velcade, thalidomide, dexamethasone) or VCD (bortezomib, cyclophosphamide, dexamethasone)], but bortezomib weekly and lower doses of steroids. ° Possible usage of combined oral therapy (ixazomib or revlimid based) ° Prolongation of therapy to 6–8 cycles followed by oral doublet if response to delay autologous transplantation • **If immediate therapy indicated for transplant ineligible:** ° Triplet {[light VRD, DRd (daratumumab + revlimid + dexamethasone), ixazomib Rd]} or doublet (Rd) with dose reduction of steroids, depending on risk and co-morbidities • Zoledronic acid every 3 months
Autologous peripheral blood stem cell transplantation	• Delay is preferred • Peripheral Blood Stem Cells mobilization with G-CSF alone
Maintenance therapy	• Lenalidomide monotherapy preferred
Relapsed/refractory	• Delay of treatment if biochemical relapse • Treatment according to standard regimens but consider using s/c and/or oral combinations

#### Introduction

Because of both humoral and cellular immunity [B, T, dendritic and natural killer (NK) cells] are impaired in MM, patients with this disease are able to easily contract SARS-Cov-2 infection and develop severe complications. Moreover, the current drugs against MM includes agents with significant hematological toxicity. Patients under treatment with immunomodulatory drugs (IMiDs) and proteasome inhibitors (PIs) are at increased risk of severe infection because of T-cell function impairment induced by these drugs. Although, anti-CD38 antibodies (daratunumab) reduce immunosuppressive regulatory T cells and deplete NK cells. Furthermore, patients with MM are older adults with possible several co-morbidities and increased risk for venous thromboembolism (33).

#### Testing for SARS-Cov-2

Testing should be performed for all newly diagnosed patients and if fever and/or other symptoms related to SARS-CoV-2 infection at any time. All patients should be tested before mobilization of peripheral blood stem cells and before autologous transplantation. Donor and recipient for allogeneic transplant should also be tested before the procedure.

#### Newly Diagnosed Patients

Specific treatment should be delayed for patients with smoldering MM or without CRAB symptoms (elevated calcium, renal failure, anemia, bone lesions), or with monoclonal gammopathy of undetermined significance (MGUS).

Patients fulfilling the CRAB criteria and/or symptoms related to multiple myeloma should be offered an initial therapy; however, treatment should be individualized to limit possible exposure to SARS-CoV-2.

For transplant-eligible patients, triplet therapy can be administered, example VRd (bortezomib, lenalidomide, dexamethasone), VCD (bortezomib, cyclophosphamide, dexamethasone), VTD (bortezomib, thalidomide, and dexamethasone); however, bortezomib doses is reduced to one weekly administration, and steroids at half-regimen doses or lower. Subcutaneous administration at home of bortezomib is preferred to limit hospital admissions. Oral triplet regimen could be used as an alternative (e.g., ixazomib, lenalidomide, dexamethazone). If stem cell transplant is differed, first line therapy could be prolonged to 6–8 cycles; then, patients could be continued on oral regimen consolidation during the SARS-CoV-2 pandemic, especially those with stable disease and with standard cytogenetic risk. Maintenance therapy can be done with lenalidomide alone (50–52).

For transplant ineligible patients, regimens like Rd (lenalidomide and dexamethasone) and DRd (daratunumab, lenalidomide and dexamethasone) could be administered depending on cytogenetic risk and comorbidities; however, doses of daratumumab should be given at a less intensive schedule. For these patients de-escalating steroids after six cycles should be considered. Oral regimens particularly ixazomib based can be given (50, 53, 54).

#### Relapse/Refractory Patients

For patients with biochemical relapse, deferment of treatment should be done.

For the remaining patients, other lines of therapy should be initiated as per standard regimens considering using s/c and oral combinations to reduce hospital attendances. Daratunumab based regimens can be administered at a less intensive schedules (50, 55, 56).

#### Hematopoietic Stem Cell Transplantation

For transplant eligible patients, delaying the stem cell transplant is considered; however peripheral stem cell collection and cryopreservation awaiting autologous transplantation could be performed after mobilization with G-CSF alone.

#### Supportive Care

Patients with a history of neutropenia and/or recurrent infections should receive G-CSF prophylactically.

Vaccination against influenza and pneumococcal species is highly recommended. Co-trimoxazole prophylaxis for pneumocystis jirovecii, antibiotics and antiherpetic prophylaxis are highly recommended.

Erythropoiesis stimulating agents should be given for severe anemia not responding to treatment. Low-molecular-weight heparin could be considered over aspirin as thromboprophlaxis.

Interval of administration of zolidronic acid should be prolonged to 3 months for all patients to limit hospital admissions.

Denosumab, when available, may be administered at home but must educate for self-administration if there is not a nursing facility.

## Lymphoid Hematological Malignancies With SARS-Cov-2 Infection (PCR Positive and/or Symptoms)

### Acute Lymphoblastic Leukemia

Systemic therapy should be delayed until resolution of the symptoms and/or PCR negativity.

Intrathecal chemotherapy can be given.

Tyrosine Kinase inhibitors can be given.

Allogeneic transplant should be deferred until resolution of symptoms and PCR negativity for at least 3–4 weeks.

A careful attention should be taken for leukemia drugs interaction with the current therapeutic options of SARS-Cov-2 infection such as drugs inducing QT prolongation, and antivirals (lopinavir/ritonavir) which can increase methotrexate and vincristine concentration.

### Chronic Lymphocytic Leukemia

For patients with no or mild symptoms, the approach is similar to those without SARS-Cov-2 infection.

For patients with more severe symptoms, monoclonal antibodies (e.g., anti CD-20) should not be used or hold if previously started.

BCR signaling inhibitors can be given.

IVIG can be given, but assessment of risks vs. benefits and close monitoring are necessary because of the higher risk of thromboembolic events with SARS-Cov-2 infection.

Allogeneic transplant should be deferred until resolution of symptoms and PCR negativity for at least 3–4 weeks.

A careful attention should be taken for drugs interaction between venetoclax and some current therapeutic options for SARS-Cov-2 infection.

### Lymphomas

If the patient has a non-severe SARS-Cov-2 infection, it is not necessary to stop specific therapy; however, administration of anti CD-20 must be avoided.

In case of severe COVID-19, specific therapy should be delayed until recovery from the infection.

Allogeneic and autologous transplantation should be deferred until resolution of the symptoms and PCR negativity for at least 3–4 weeks.

### Multiple Myeloma

Patients with no symptoms of COVID-19, but with symptoms related to MM who need immediate therapy (acute renal failure, heavy anemia, extended bone disease, etc.), should start treatment, or continue if it has been started, particularly if no optimal response is obtained. However, steroids and drugs inducing lymphopenia should be de-intensified.

Patients with symptoms of COVID-19 should not be treated immediately or should interrupt specific therapy until resolution of the symptoms and PCR negativity. Steroids should be tapered to zero.

Autologous and allogeneic transplantation should not be performed.

A careful attention should be taken for significant interaction between anti myeloma therapy and therapeutic options for COVID-19 such as hydroxychloroquine, azithromycin and antivirals.

## Data Availability Statement

The raw data supporting the conclusions of this article will be made available by the authors, without undue reservation.

## Author Contributions

AI major contributors in writing the manuscript. PN contributed in data collection. AT contributed in data collection and analysis. CK contributed in writing the manuscript and data collection. All authors contributed to the article and approved the submitted version.

## Conflict of Interest

The authors declare that the research was conducted in the absence of any commercial or financial relationships that could be construed as a potential conflict of interest.
